# Long-term Effectiveness, Treatment Persistence, and Timing of Virological Failure With Long-Acting Cabotegravir Plus Rilpivirine in Real-World HIV Care: RELATIVITY Cohort

**DOI:** 10.1093/ofid/ofag622

**Published:** 2026-10-21

**Authors:** Luis Buzón, Noemí Cabello-Clotet, María Luisa Montes, María José Galindo, Rosa María Martínez, Mireia Santacreu, María Remedios-Alemán, Miguel Torralba, Francisco Fanjul, Manuel Gutiérrez-Cuadra, Adrián Rodríguez, María José Crusells, María Navarro, María Aguilera-García, Carmen Hidalgo-Tenorio, Luis Enrique Morano, David Vinuesa, Enrique Bernal-Morell, Carlos de Andrés, Hadrián Pernas, Juan Tiraboschi, Alejandro Bendala, María Jesús Vivancos, Cristina Díez, Guillermo Soria, Desiré Pérez, Mireia Cairó, Alberto Romero, Rebeca Cabo, Josefa Soler, María Antonia Sepúlveda, Joaquín Bravo, Cristina Escrich, Eva Ferreira, Beatriz Valentín, Magdalena Muelas, Ana Lucas-Dato, Albert Gómez, Ana Cerezales, Juan Emilio Losa, José Sanz, Mar Masía, Juan José Corte-García, Ana Antolí, Juan Carlos Gainzarain, Miriam Estébanez, Patricia Barragán, Marta Clavero, Óscar Ferrero, Jesús Troya, RELATIVITY Cohort, Ana Cristina Antolí Royo, Carmen Grande Sáez, María Ángeles Garcinuño Jiménez, Ana Carrero Gras, Eva María Ferreira Pasos, Hadrian Pernas Pardavila, Mercedes González, Nuria Vázquez Temprano, Miriam Estébanez, Tatiana Mata-Forte, Ana Muñoz Gómez, Claudia Dorta Hernández, José Reynaldo Homen Fernández, Juncal Pérez-Somarriba Moreno, Maravillas Carralón, María José Núñez Orantos, María Nieves Sanz Pérez, Noemí Cabello-Clotet, Philip Erick Wickman Jorgensen, Susana Olmedo Hernández, Teresa Martínez Burgoa, Valeria Cabral Sousa, Vicente Estrada Pérez, Virginia Víctor Palomares, Ana Ferrer Ribera, Andreu Belmonte, Ángela Pradilla Dieste, Carolina Pinto, María José Galindo Puerto, Rosa Oltra Sempere, Sandra Pérez Gómez, Álvaro Cecilio, Isabel Sanjoaquín Conde, María José Crusells Canales, Pilar Jiménez Marcen, Ana Lucas Dato, Belén Martínez López, Inmaculada González Cuello, Jara Llenas García, María García López, Carolina Navarro, Luis Buzón Martín, María Fernández, Amparo Exojo Morales, Carlos de Andrés David, Inmaculada Poquet Catalá, Karenina Antelo Cuéllar, Laia Navarro Peiró, María Josefa de la Asunción Villaverde, Silvia Lope Bolumar, Alba León Barbosa, Esther Nogales Nieves, Eva García Alcalde, Juan José Corte García, Lucía Alonso Alonso, María Vanessa López Peláez, Sara Jaber Carballo, Albert Gómez Lozano, Sara García Torras, Marta Milián Sanz, Ana Isabel Lérida Urteaga, Antonia Pérez Barja, Javier López-Nieto Sempere, Magdalena Muelas Fernández, Beatriz de la Calle, Ángela Botella Zaragoza, Félix Gutiérrez Rodero, Javier García Abellán, Mar Masiá, Paula Mascarell Arlandis, Sergio Padilla Urrea, Chiara Fanciulli, Cristina Díez Romero, Francisco Tejerina Picado, Juan Carlos López Bernaldo de Quirós, Leire Pérez Latorre, María Teresa Aldamiz-Echevarría Lois, Antonio Jesús Sánchez Guirao, Ángeles Muñoz Pérez, Antonia Alcaraz García, Cristina Tomás Jiménez, Elena Guijarro Westermeyer, Enrique Bernal Morell, Eva García Villalba, Joaquín Bravo Urbieta, María Dolores Hernández Lorente, Rodrigo Martínez Rodríguez, Román González Hipólito, Noemí Patricia Barragán Gallo, Alberto Juárez Toquero, Beatriz Valentín Casado, Jesica Abadía Otero, Julia Gómez Barquero, Pablo Bachiller Luque, Yolanda Arranz García, Adriana Pinto, David Rial, Federico Pulido, Juan Martín, Laura Bermejo Plaza, María de Lagarde, María Teresa López Caballero, Mireia Santacreu, Otilia Bisbal, Rafael Rubio, Roser Navarro, Victoria Macheño del Real, Celia Miralles Álvarez, Guillermo Pousada, Henar de las Heras Miralles, Luis Enrique Morano Amado, Víctor Asensi, Laura Guio Carrión, Mikel del Álamo, Ester Sáez de Adana Arroniz, Juan Carlos Gainzarain Arana, Ana Vegas Serrano, Juan Emilio Losa García, María Velasco Arribas, Maite Ganchegui Aguirre, Miriam López Martínez, Óscar Luis Ferrero Benítez, Alicia Sedo Mor, Analuz Fernández, Juan Tiraboschi, Josefa Soler González, Víctor Arenas García, Lucía Romero, Nereyda Tosco García, Remedios Alemán Valls, Guillermo Soria Fernández-Llamazares, Juan Víctor San Martín López, Ruth Calderón Hernáiz, Alberto Delgado Fernández, Lorenzo Sánchez, Miguel Torralba González de Suso, Alberto Romero Palacios, María Antonia Sepúlveda, Alejandra Gimeno García, Alejandro David Bendala Estrada, Carmen Montero Hernández, Diana Corps Fernández, Jose Carlos Escribano, María del Mar García Navarro, Marouane Menchi Elanzi, Ana Cerezales Calviño, Bárbara Alonso Moreno, José Fernando Lluch Perales, Alfonso Cabello, Miguel Górgolas Hernández-Mora, María Rubio Olivera, Marta Clavero Olmos, Belén Escribano Losada, Guillermo Cuevas, Jesús Troya García, Mariano Matarranz, Pablo Ryan, Sandra Rodríguez Perut, Alejandro de Gea, Ana Belén Delgado Hierro, Carmen Busca, José Ignacio Bernardino, Juan González García, Luz Martín Carbonero, María del Mar Arcos Rueda, María Luisa Montes, Rafael Micán, Rocío Montejano, Andoni Casen Gil, Ignacio de los Santos, María Aguilera García, Aitziber Illaro Uranga, Carlos Armiñanzas Castillo, Claudia González-Rico, Francisco Arnaiz de las Revillas Almajano, Manuel Gutiérrez Cuadra, María Carmen Fariñas Álvarez, Noelia Ruiz-Alonso, Paula Runza, Raúl Parra-Fariñas, Ángela Forcén Vicente de Vera, María Aranzazú Caudevilla Martínez, Rosa María Martínez Álvarez, Ruth Caballero Asensio, David Dalmau Juanola, Laura Gisbert Pérez, Mireia Cairó Llobell, Roser Font Canals, Xavier Martínez Lacsa, José Sanz, Alberto Díaz de Santiago, Ana Fernández Cruz, Sara de la Fuente Moral, Ana Moreno, Daniel de las Heras Gómez, María Jesús Pérez Elías, María Jesús Vivancos Gallego, Mario Pons Guillén, Desiré Pérez Martínez, María Elisa Pino Díaz, Miguel Alberto de Zárraga Fernández, Miguel Vicente Egido Murciano, Teresa Omiste Sanvicente, Adrián Ferré, Antoni Campins, Francisco Fanjul, Luisa Martín Pena, Melchor Riera, Adrián Rodríguez, Patricia Sorní Moreno, Carmen Hidalgo Tenorio, Sergio Sequera Arquelladas, Cristina Escrich, María del Carmen Navarro, David Vinuesa, Noemí Cabello-Clotet, Paula Runza, David Vinuesa, Miguel Torralba, Francisco Fanjul, Adrián Rodríguez, Sonia Calzado, Luis Enrique Morano, Hadrián Pernas, Enrique Bernal

**Affiliations:** Infectious Diseases Division, Hospital Universitario de Burgos, Burgos, Spain; Infectious Diseases Division, Hospital Universitario Clínico San Carlos, Madrid, Spain; Infectious Diseases Division, HIV Unit, Hospital Universitario La Paz, Madrid, Spain; Infectious Diseases Division, Hospital Clínico Universitario de Valencia, Valencia, Spain; Infectious Diseases Division, Hospital Universitario Miguel Servet, Zaragoza, Spain; Infectious Diseases Division, HIV Unit, Hospital Universitario 12 de Octubre, Madrid, Spain; Infectious Diseases Division, Hospital Universitario de Canarias, Santa Cruz de Tenerife, Spain; Infectious Diseases Division, Internal Medicine, Hospital Universitario de Guadalajara, Guadalajara, Spain; Infectious Diseases Division, Hospital Universitari Son Espases, Palma de Mallorca, Spain; Infectious Diseases Department, Hospital Universitario Marqués de Valdecilla, Santander, Spain; Instituto de Investigación Sanitaria de les Illes Balears, Hospital Universitario Son Llátzer, Palma de Mallorca, Spain; Infectious Diseases Department, Hospital Clínico Universitario Lozano Blesa, Zaragoza, Spain; Infectious Diseases Department, Consorci Corporació Sanitària Parc Taulí, Sabadell, Spain; Infectious Diseases Division, Internal Medicine, Hospital Universitario La Princesa, Madrid, Spain; Infectious Diseases Division, Hospital Universitario Virgen de las Nieves, Granada, Spain; Infectious Diseases Division, Hospital Universitario Álvaro Cunqueiro, Vigo, Pontevedra, Spain; Infectious Diseases Division, Hospital Universitario Clínico San Cecilio, Granada, Spain; Infectious Diseases Division, Hospital General Universitario Reina Sofía, Murcia, Spain; Infectious Diseases Division, Hospital de Dénia-Marina Salud, Alicante, Spain; Infectious Diseases Division, Complexo Hospitalario Universitario de Pontevedra, Pontevedra, Spain; Infectious Diseases Department, Hospital Universitario de Bellvitge, Barcelona, Spain; Internal Medicine, Hospital Universitario de Torrejón, Madrid, Spain; Infectious Diseases Department, Hospital Universitario Ramón y Cajal, Madrid, Spain; Infectious Diseases Department, Hospital General Universitario Gregorio Marañón, Madrid, Spain; Infectious Diseases Division, Internal Medicine, Hospital Universitario de Fuenlabrada, Fuenlabrada, Spain; Infectious Diseases Division, Hospital Universitario San Agustín, Avilés, Spain; Infectious Diseases Department, HIV Unit, Hospital Universitari Mútua Terrassa, Terrassa, Spain; Infectious Diseases Division, Hospital Universitario de Puerto Real, Puerto Real, Cádiz, Spain; Infectious Diseases Division, Hospital Universitario Central de Asturias, Oviedo, Spain; Infectious Diseases Division, Internal Medicine, Hospital Universitario de Cabueñes, Gijón, Spain; Infectious Diseases Division, Hospitalario Universitario de Toledo, Toledo, Spain; Infectious Diseases Division, Hospital General Universitario Morales Meseguer, Murcia, Spain; Infectious Diseases Division, Hospital Verge de la Cinta, Tortosa, Spain; Internal Medicine, Complejo Asistencial de Segovia, Segovia, Spain; Infectious Diseases Division, Internal Medicine, Hospital Universitario Río Hortega, Valladolid, Spain; Internal Medicine, Hospital de Viladecans, Viladecans, Spain; Infectious Diseases Division, Hospital Comarcal de la Vega Baja, Alicante, Spain; Infectious Diseases Division, Hospital de Santa Caterina de Salt, Girona, Spain; Internal Medicine, Hospital Universitario Doctor José Molina Orosa, Las Palmas, Spain; Infectious Diseases Department, Hospital Universitario Fundación de Alcorcón, Alcorcón, Spain; Infectious Diseases Division, Hospital Universitario Príncipe de Asturias, Alcalá de Henares, Spain; Infectious Diseases Division, Hospital General Universitario de Elche, Elche, Spain; Pharmacy Department, Hospital de Jove, Gijón, Spain; Internal Medicine, Complejo Universitario Asistencial de Ávila, Ávila, Spain; Infectious Diseases Department, Hospital Universitario de Álava, Vitoria, Spain; Infectious Diseases Division, NRBQ Unit, Hospital Central de la Defensa Gómez Ulla, Madrid, Spain; Internal Medicine, Hospital Residencia Sant Camil, Sant Pere de Ribes, Barcelona, Spain; Infectious Diseases Unit, Hospital Universitario Infanta Elena, Valdemoro, Madrid, Spain; Infectious Diseases Department, Hospital Universitario Basurto, Bilbao, Spain; Infectious Diseases Division, Internal Medicine, Hospital Universitario Infanta Leonor, Madrid, Spain

**Keywords:** cabotegravir, HIV, long-acting injectable, rilpivirine, virological failure

## Abstract

**Background:**

Long-acting injectable cabotegravir plus rilpivirine (CAB + RPV LA) is an established maintenance option for virologically suppressed people with human immunodeficiency virus-1 (HIV-1), but long-term real-world persistence and virological failure (VF) data remain limited.

**Methods:**

RELATIVITY is an ongoing nationwide ambispective multicenter cohort of adults with HIV who switched from suppressive oral antiretroviral therapy to CAB + RPV LA in routine care in Spain. Data through 1 March 2026 were analyzed. The outcomes were virological suppression, treatment persistence, protocol-defined VF, and time to discontinuation-related events. Virological failure was defined as 2 consecutive HIV-1 RNA values ≥200 copies/mL or 1 value ≥500 copies/mL prompting discontinuation.

**Results:**

Among 3349 participants, 3343 had valid follow-up dates; the median follow-up was 25.1 months (19.3–30.8). Virological suppression was documented in 3197/3349 (95.5%) overall and 3197/3309 (96.6%) with an evaluable closest viral load; 40 (1.2%) lacked an evaluable value. Injection adherence was ≥90% in 3257/3280 (99.3%) evaluable participants. Overall, 2979/3349 (89.0%) remained on CAB + RPV LA. Virological failure occurred in 28/3349 (0.8%); 20/28 (71.4%) occurred within the first 12 months after switching. Emergent integrase strand transfer inhibitor and/or nonnucleoside reverse transcriptase inhibitor resistance was detected in 19/28 VF cases, and 24/28 achieved virological suppression after switching to oral therapy. Treatment-limiting injection-site reactions and systemic adverse events occurred in 2.4% and 1.6%, respectively.

**Conclusions:**

Long-acting injectable cabotegravir plus rilpivirine showed high virological suppression and persistence beyond 2 years in routine care. Virological failure was uncommon and clustered mainly within 12 months, supporting early virological monitoring and prompt resistance-guided management.

## Teaser

Among 3349 virologically suppressed adults, long-acting cabotegravir plus rilpivirine maintained high suppression and treatment persistence over a median of 25.1 months. Virological failure was uncommon, clustered mainly within 12 months, and frequently involved emergent resistance among the 28 cases.

Long-acting injectable cabotegravir plus rilpivirine (CAB + RPV LA) is an alternative to daily oral antiretroviral therapy (ART) in virologically suppressed people with human immunodeficiency virus (PWH). However, its long-term performance in routine care requires evaluation beyond trial efficacy, particularly regarding treatment persistence, virological failure (VF), and resistance [1–5].

Randomized trials, including ATLAS, FLAIR, SOLAR, CARES, LATTE-2, and ATLAS-2M, have demonstrated noninferior efficacy, durable virological suppression, and favorable safety and acceptability in selected populations [1–9].

Real-world populations differ from trial populations in treatment history, historical genotype availability, comorbidities, body mass index, and injection logistics. Archived resistance, pharmacokinetic exposure, injection technique, and visit timing may affect outcomes, and VF can be accompanied by emergent integrase strand transfer inhibitor (INSTI) and/or nonnucleoside reverse transcriptase inhibitor (NNRTI) resistance [10, 11]. Observational cohorts complement trials by assessing effectiveness and persistence under less standardized routine-care conditions [12–14].

The timing of the VF is also clinically relevant. Previous analyses suggest that events may cluster early after switching rather than accumulate steadily, with implications for viral load monitoring, resistance testing, and subsequent ART selection [2, 10, 11, 15].

We evaluated long-term virological suppression, treatment persistence, discontinuation patterns, timing, and resistance consequences of VF, injection adherence among VF cases, and outcomes after return to oral ART in the Spanish RELATIVITY cohort.

## METHODS

### Study Design and Setting

The RELATIVITY study is an ongoing, nationwide, multicenter, uncontrolled cohort of PWH who switched from suppressive oral ART to CAB + RPV LA in routine care in Spain.

This analysis used data until 1 March 2026. Individuals who initiated CAB + RPV LA between 1 June 2023 and 31 December 2024 were retrospectively included and followed prospectively. Fifty-eight centers joined the cohort, and 53 contributed to this update after 5 centers (290 participants) were temporarily excluded because the outcomes had not been fully updated since the previous published analysis [13]. This restriction reduced the bias from partial site updates. Clinical care followed local protocols. RELATIVITY is registered at ClinicalTrials.gov (NCT07703748).

### Study Population

Adults (≥18 years) were eligible for the primary cohort if they had HIV-1 RNA <50 copies/mL while receiving oral ART and received at least one intramuscular CAB + RPV LA dose before 31 December 2024 [13].

Protocol-level eligibility criteria at CAB + RPV LA initiation required suppressed viremia and excluded pregnancy or breastfeeding, prior CAB + RPV LA trial participation, hepatitis B surface antigen (HBsAg) positivity, and known resistance-associated mutations (RAMs) to rilpivirine or second-generation INSTI RAMs expected to compromise activity; isolated K103N was permitted [16, 17]. These criteria were not reapplied as post hoc analytical exclusions in this update: 6 analyzed records were coded as active hepatitis B and were retained. Missing values for a particular outcome were handled as outcome-specific nonevaluability rather than cohort-level exclusion. Supplementary Table 1 describes a stricter on-label sensitivity subgroup that excluded historical NNRTI/INSTI RAMS and prior VF involving those classes.

### Outcomes

The primary outcomes were virological suppression, treatment persistence, and protocol-defined VF. Virological suppression was HIV-1 RNA <50 copies/mL at the closest evaluable measurement to database lock. Persistence was defined as no permanent CAB + RPV LA discontinuation at the last documented follow-up; temporary oral bridging or interruption did not indicate nonpersistence if injections resumed. Virological failure was defined as 2 consecutive HIV-1 RNA values ≥200 copies/mL or 1 value ≥500 copies/mL prompting discontinuation; the discontinuation qualifier “leading to treatment discontinuation” applied only to the single-measurement ≥500 copies/mL prompting discontinuation; the discontinuation qualifier applied only to the single-value criterion. Suspected rebounds were centrally adjudicated. Viral blips were defined as values of 50 to <200 copies/mL followed by resuppression without treatment modification. The 2 consecutive ≥200 copies/mL component matched OPERA, whereas the single-value criterion differed from OPERA (≥200 copies/mL plus discontinuation) and CONSENSUS-LAI (>1000 copies/mL or other consensus conditions); the overall definition was therefore only partially concordant [12, 18].

Secondary outcomes included permanent discontinuation and its recorded reasons, treatment-limiting adverse events, immunological changes, injection adherence, emergent resistance in VF cases, and suppression after returning to oral ART.

### Injection Adherence Assessment

Injection adherence was assessed from administered versus scheduled injection dates. The proportion of days covered (PDC) was the days covered by CAB + RPV LA, including the ±7-day window, divided by the follow-up days and categorized as 100%, 90%–99.9% or <90%. PDC measured visit/injection timing, not pharmacokinetic exposure or injection technique. Participants lacking complete scheduled-administered date pairs were nonevaluable.

### Data Collection

Baseline demographic and clinical data were obtained from electronic medical records and laboratory databases. Prespecified VF-case variables included age, sex, country/region of birth, body mass index, immediate preswitch ART, previous VF, historical genotype/subtype, baseline RAMs, HIV-1 RNA, timing at VF, emergent RAMs, injection timing, post-VF ART, and resuppression. Follow-up testing followed local practice. Safety data were limited to adverse events prompting oral bridging, temporary interruption, or permanent discontinuation; other adverse events were not systematically collected. Pregnancy was not a dedicated longitudinal variable; although pregnancy or pregnancy intention could be recorded when it prompted treatment change. Data were entered in REDCap and merged into an anonymized database [19, 20].

### Statistical Analysis

Continuous variables are summarized as medians (interquartile range), and categorical variables as frequencies and percentages. Kaplan–Meier methods described time to VF and discontinuation-related events, with censoring at the last follow-up or database lock. Reason-specific curves were descriptive because reasons could overlap and were not competing-risk cumulative-incidence estimates; events without evaluable dates were omitted. Suppression was reported using an evaluable denominator and the full cohort, in which missing closest viral loads counted as no documented suppression. Missing data were not imputed. Paired immunological changes at 12 and 24 months and the last assessment were tested with the Wilcoxon signed-rank test. No multivariable modeling was performed because analyses were descriptive. R version 4.5.3 was used.

### Ethical Considerations

The local Ethics Committee approved the study (code 2596), which was communicated to the Spanish Agency for Medicines and Health Products (23-00144). Data were anonymized and handled in accordance with national data protection and ethical standards.

## RESULTS

### Cohort Overview and Follow-up

The cohort included 3349 virologically suppressed individuals from 53 centers in Spain. In the previously published parent RELATIVITY analysis, 3203 treated individuals were screened, and 57 were excluded for detectable HIV-1 RNA at switching [13]. This update includes only participants with documented baseline suppression; counts for other protocol-level screen failures were not retained. Five centers (290 participants) were temporarily excluded because follow-up updates were incomplete. Baseline characteristics are presented in Table 1. Immediate preswitch regimens were heterogeneous; 2592/3349 (77.4%) included an INSTI.

**Table 1.** Baseline Patient Demographic and Clinical Characteristics

Variables	Global Cohort (N = 3349)
Age, y	45.0 (37.0–54.0) (n = 3349)
Body mass index, kg/m^2^	25.4 (23.2–28.1) (n = 2875)
Gender, n (%)	
Female	501/3349 (15.0%)
Male	2825/3349 (84.4%)
Transgender male	0/3349 (0.0%)
Transgender female	23/3349 (0.7%)
Nationality, n (%)	
Spanish	2331/3338 (69.8%)
Other	1007/3338 (30.2%)
HIV transmission route, n (%)	
MSM	1889/3349 (56.4%)
Heterosexual	566/3349 (16.9%)
IDU	223/3349 (6.7%)
Vertical	28/3349 (0.8%)
Other	212/3349 (6.3%)
Not available	431/3349 (12.9%)
Immunological and virological characteristics, n	
CD4 nadir, cells/mm^3^	339.0 (197.0–499.0) (n = 2681)
Baseline CD4+ T-cell, cells/mm^3^	797.0 (601.5–1030.5) (n = 3059)
Baseline CD4+ T-cell percentage, %	35.0 (29.0–41.2) (n = 2829)
Baseline CD8+ T-cell, cells/mm^3^	825.8 (606.0–1104.0) (n = 3006)
Baseline CD4/CD8 ratio	1.0 (0.7–1.3) (n = 3006)
HIV-1 RNA at diagnosis, copies/mL	54 685.0 (14 950.0–184 042.2) (n = 2272)
Time from diagnosis to start of first ART, mo	2.0 (0.5–19.0) (n = 3106)
History of AIDS, n (%)	
Yes	410/3349 (12.2%)
No	2611/3349 (78.0%)
Not available	328/3349 (9.8%)
Time from start of ART to switch, years	9.0 (5.0–15.0) (n = 3246)
Duration of virological suppression before switch, mo	81.5 (33.0–132.0) (n = 2660)
Previous virological failure on any ART regimen, n (%)	
Yes	108/3349 (3.2%)
No	2598/3349 (77.6%)
Not available	643/3349 (19.2%)
Third drug involved in the virological failure, n (%)	
INSTI	23/108 (21.3%)
NNRTI	26/108 (24.1%)
PI	38/108 (35.2%)
Not available	21/108 (19.4%)
Historical genotype available, among participants with recorded genotype availability status, n/N (%)	1540/3235 (47.6%)
HIV-1 subtype B	688/1540 (44.7%)
HIV-1 subtype A1/A2	34/1540 (2.2%)
HIV-1 subtype F/CRF	46/1540 (3.0%)
Other HIV-1 subtypes	66/1540 (4.3%)
HIV-1 subtype not available	706/1540 (45.8%)
Wild-type virus without mutations, n (%)	1319/1540 (85.6%)
Virus harboring RAMs to NRTI, n (%)	123/1540 (8.0%)
M184V	25/1540 (1.6%)
Others	108/1540 (7.0%)
Virus harboring RAMs to NNRTI, n (%)	123/1540 (8.0%)
K103N	48/1540 (3.1%)
E138A	13/1540 (0.8%)
Others	74/1540 (4.8%)
Virus harboring RAMs to INSTI, n (%)	12/1540 (0.8%)
L74M/I/F	1/1540 (0.1%)
T97A	1/1540 (0.1%)
Others	10/1540 (0.6%)
Number of resistance mutations, n (%)	
No mutations detected	1319/1540 (85.6%)
1 mutation detected	184/1540 (11.9%)
2 mutations detected	37/1540 (2.4%)
3 mutations detected	0/1540 (0.0%)
Reasons for switching to CAB + RPV LA, n (%)	
Toxicity	43/3349 (1.3%)
Drug interaction	11/3349 (0.3%)
Simplification	795/3349 (23.7%)
Convenience/quality of life	1676/3349 (50.0%)
Malabsorption	40/3349 (1.2%)
Swallowing disorders	14/3349 (0.4%)
Patient request	1253/3349 (37.4%)
Other reasons for change	324/3349 (9.7%)
Oral lead-in before injectable treatment, n (%)	404/3349 (12.1%)
Follow-up time, months	25.1 (19.3–30.8) (n = 3343)
Effectiveness	
Closest viral load <50 copies/mL, full-cohort denominator, n/N (%)	3197/3349 (95.5%)
Closest viral load ≥50 copies/mL, n/N (%)	112/3349 (3.3%)
No evaluable closest viral load, n/N (%)	40/3349 (1.2%)
Treatment status at end of follow-up, n (%)	
Temporary oral bridging or interruption	71/3349 (2.1%)
Permanent discontinuation	370/3349 (11.0%)
Persistence on treatment	2979/3349 (89.0%)
Reasons for treatment discontinuation or interruption, n (%)	
Systemic adverse events	54/3349 (1.6%)
Injection-site reactions	79/3349 (2.4%)
Virological failure	28/3349 (0.8%)
Other reasons	248/3349 (7.4%)
Injection adherence (PDC) ≥90%, among evaluable participants, n/N (%)	3257/3280 (99.3%)
Injection-schedule data not evaluable, n/N (%)	69/3349 (2.1%)

Data are median (IQR) unless otherwise stated. Multiple reasons for switching or discontinuation/interruption could be recorded, and oral bridging or temporary interruption was not mutually exclusive with persistence. Mutation-specific rows may overlap. Genotype availability was ascertainable in 3235/3349 participants, closest viral load in 3309/3,349, and PDC in 3280/3349; missing data reflect unavailable source information.

Abbreviations: AIDS, acquired immunodeficiency syndrome; ART, antiretroviral therapy; CAB, cabotegravir; IDU, injection drug use; INSTI, integrase strand transfer inhibitor; IQR, interquartile range; LA, long-acting; MSM, men who have sex with men; NNRTI, nonnucleoside reverse transcriptase inhibitor; NRTI, nucleoside reverse transcriptase inhibitor; PDC, proportion of days covered; PI, protease inhibitor; RAMs, resistance-associated mutations; RPV, rilpivirine; VF, virological failure; VL, viral load.

At database lock, the median follow-up among 3343 participants with evaluable dates was 25.1 months (interquartile range [IQR], 19.3–30.8). Overall, 2979/3349 (89.0%) remained on CAB + RPV LA and 370/3349 (11.0%) permanently discontinued treatment. Among those who persisted, 2908 continued without recorded interruption, and 71 had temporary oral bridging or interruption. Detailed stratification according to the strict on-label criteria for CAB + RPV LA use is provided in Supplementary Table 1.

### Virological Suppression

Virological suppression at the closest assessment was documented in 3197/3349 (95.5%) using the full cohort. Among 3309 participants with an evaluable closest viral load, 3197 (96.6%) had HIV-1 RNA <50 copies/mL and 112 (3.4%) had ≥50 copies/mL; 40/3349 (1.2%) lacked an evaluable result. Thus, the former combined group of 152 comprised 112 with HIV-1 RNA ≥50 copies/mL and 40 with a missing assessment.

### Injection Adherence

Among 3280 participants with evaluable injection schedules, 3257 (99.3%) had PDC ≥90% (2309 at 100% and 948 at 90%–99.9%), and 23 (0.7%) had PDC <90%. Proportion of days covered was nonevaluable for 69 participants because scheduled-administered date pairs were incomplete.

### Treatment Persistence

Permanent discontinuation occurred in 370/3349 participants (11.0%). The recorded reasons or events were other nonvirological reasons in 248 (7.4%), injection-site reactions in 79 (2.4%), systemic adverse events in 54 (1.6%), and VF in 28 (0.8%) (Figure 1). Categories could overlap and are not mutually exclusive patient groups.

**Figure 1.**
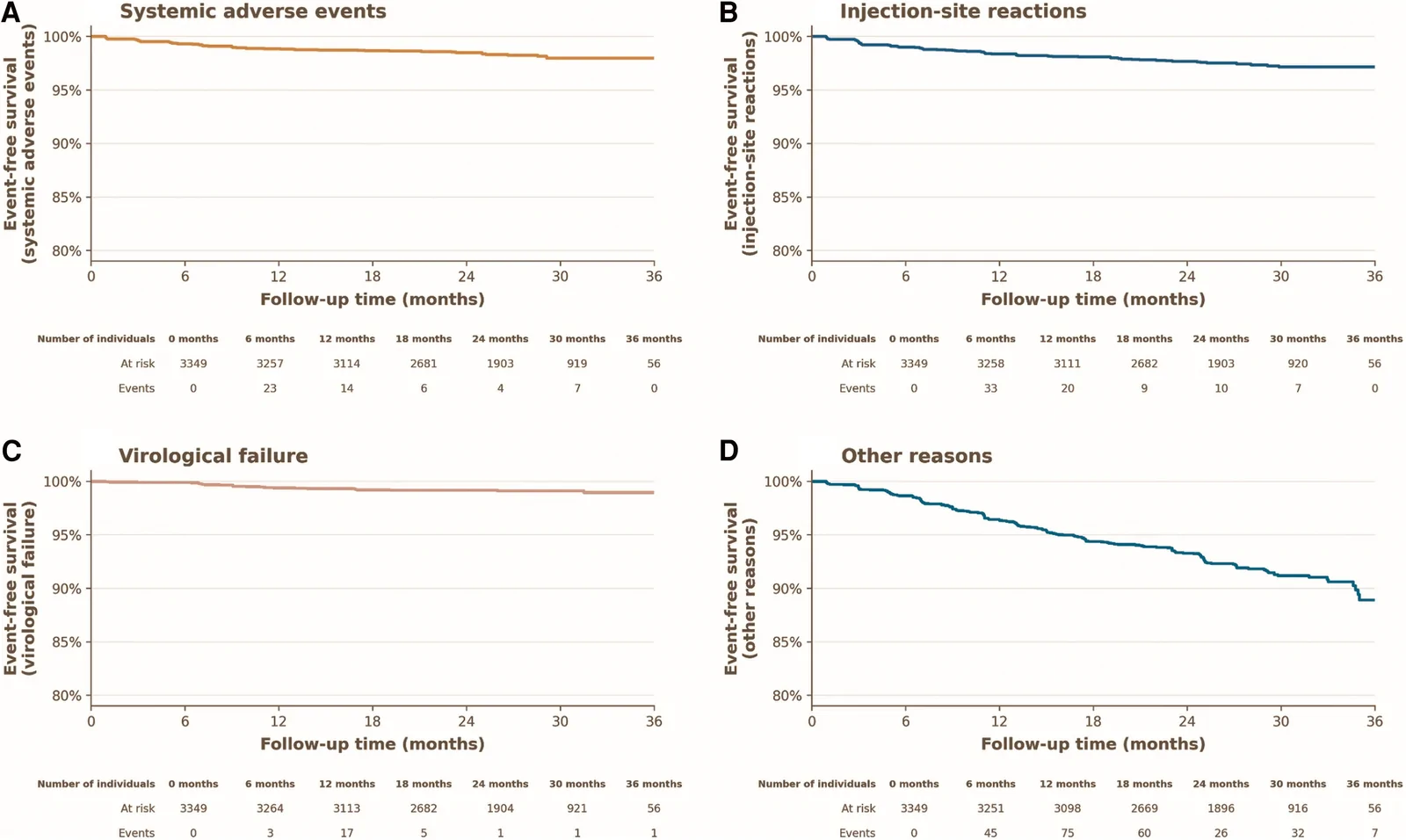
Kaplan–Meier estimates of event-free survival for discontinuation-related outcomes. Panels A–D show systemic adverse events, injection-site reactions, virological failure, and other reasons, respectively. Risk sets are event-specific; categories may overlap, and curves are descriptive rather than competing-risk estimates. Of the 248 participants with an “other reason,” 245 had evaluable event times within 36 mo.

Other reasons included personal decisions, administrative or logistical barriers, transfer, relocation, and loss to follow-up. Continued CAB + RPV LA use outside the participating network was not systematically ascertainable after transfer, relocation, or loss to follow-up; therefore, some participants may have continued elsewhere.

Treatment-limiting adverse events and VF occurred mainly earlier, whereas other reasons arose throughout the follow-up; no formal accumulation rate was calculated. Virological failure timing is detailed below.

### Virological Failure: Clinical Characteristics and Timing

Protocol-defined VF occurred in 28/3349 (0.8%) (Table 2). Twenty-one (75.0%) were male; the median age was 42.0 years (IQR, 34.0–50.5), and the median body mass index (BMI) was 28.4 kg/m^2^ (IQR, 24.8–30.8), with 9/28 (32.1%) at ≥30 kg/m^2^. At discontinuation, 24/28 had HIV-1 RNA ≥500 copies/mL, and 4 had 200–499 copies/mL after 2 consecutive values ≥200 copies/mL. Isolated HIV-1 RNA values ≥500 copies/mL without discontinuation were not separately enumerated or classified as VF under the single-value rule.

**Table 2.** Characteristics and Outcomes of PWH Who Experienced Virological Failure During Long-acting Cabotegravir Plus Rilpivirine Treatment

Patient	Sex	Age, y	Country/Region of Birth	BMI, kg/m^2^	Previous Treatment	Month of VF-related Discontinuation	Viral Load at Discontinuation, Copies/mL	Previous RAMs/Subtype	New RAMs (INSTI)	New RAMs (NNRTI)	Oral ART After VF	HIV-1 RNA <50 copies/mL After Oral Treatment
1	Male	48	Portugal	26.7	DTG/3TC	9.9	43 300	Wild type/F-CRF	E138K, Q148K	No	DRV/c/FTC/TAF	Yes
2	Male	57	Spain	24.9	RAL + FTC/TAF	11.0	3236	M184V/NA	No	No	DTG + DOR + 3TC	Yes
3	Male	34	Romania	19.3	BIC/FTC/TAF	9.1	1692	Not available	E138K, G140GS, Q148K/R	No	DTG + FTC/TAF	Yes
4	Female	23	Guinea Equatorial	28.7	BIC/FTC/TAF	13.1	82 226	Not available	No	E138A, M230L	DRV/c/FTC/TAF^a^	Yes
5	Male	34	Spain	27.7	DTG/RPV	16.9	868	Not available	N155H	E138K	DRV/c + ABC/3TC	Yes
6	Male	50	Colombia	33.9	DTG/3TC	9.0	128 000	Wild type/NA	L74M, T97A, G140S, Q148K/R, E157Q	L100I, K103N	DRV/c/FTC/TAF	Yes
7	Male	49	Peru	22.5	BIC/FTC/TAF	7.1	3140	Wild type/B	No	No	DRV/c/FTC/TAF^a^	Yes
8	Male	45	Angola	29.3	DTG/3TC	6.9	44 000	Not available	E138K, Q148R, L74L/M	K103N, Y188L	DRV/c/FTC/TAF	Yes
9	Male	30	Venezuela	31.5	DTG/3TC	19.1	1720	Wild type/B	No	No	BIC/FTC/TAF	Yes
10	Male	46	Spain	23.4	BIC/FTC/TAF	8.2	290	Wild type/NA	Q148R	No	BIC/FTC/TAF	Yes
11	Female	52	Spain	36.4	DTG/RPV	1.2	362	M184V; K103N/B	No	No	DRV/c/FTC/TAF^a^	Yes
12	Male	55	Spain	29.1	BIC/FTC/TAF	9.0	7100	Wild type/F-CRF	Q148R, G163R	E138A, V179E, P225H, F227L, N348I	DRV/c/FTC/TAF	Yes
13	Male	28	Venezuela	28.2	BIC/FTC/TAF	0.9	7154	Not available	No	No	DRV/c/FTC/TAF^a^	Yes
14	Male	58	Spain	29.2	DTG/RPV	16.8	512	Wild type/NA	No	No	BIC/FTC/TAF	No
15	Female	41	Paraguay	39.6	DTG/RPV	3.0	31 000	Wild type/B	No	No	DRV/c/FTC/TAF^a^	Yes
16	Male	29	Venezuela	28.4	BIC/FTC/TAF	7.1	263 861	Not available	L74L/M, E138K, Q148K/R	K101P, K103S	DRV/c/FTC/TAF	Yes
17	Male	43	Spain	23.4	BIC/FTC/TAF	11.0	2331	Wild type/B	Q146L	K101E	BIC/FTC/TAF	Yes
18	Female	47	Dominican Republic	30.6	BIC/FTC/TAF	7.2	12 147	Wild type/B	No	K101K/E, V106V/I, Y181C	BIC/FTC/TAF	Yes
19	Female	33	Peru	38.3	BIC/FTC/TAF	6.9	5158	Not available	N155H, E157Q	K101E	DRV/c/FTC/TAF	Yes
20	Female	59	Spain	23.7	BIC/FTC/TAF	6.4	18 580	Not available	G140S, L74M, Q148H	No	DRV/c/FTC/TAF	Yes
21	Male	58	Spain	28.3	DTG/RPV	11.1	10 500	Wild type/F-CRF	No	K103N, N348I, Y181C	BIC/FTC/TAF	Yes
22	Male	40	Spain	24.3	DTG/ABC/3TC	6.9	289	Wild type/B	No	No	BIC/FTC/TAF	Yes
23	Male	36	Venezuela	31.4	DTG/RPV	31.5	435	Not available	N155H, L74M, T97A	K101E, E138K, M230L	DRV/r + FTC/TAF	Yes
24	Female	21	South America	18.6	DTG/3TC	26.0	3510	Wild type/NA	E138K, S147G, N155H, R263K	No	DOR + FTC/TDF	No
25	Male	62	Spain	25.1	DTG/3TC	9.0	115 000	Not available	E138K, G140A, Q148R	K103N, E138K, N348I	DRV/c/FTC/TAF	Yes
26	Male	41	Spain	36.4	DTG/RPV	10.8	229 000	Not available	G140, Q148R	S68G, K101E	DRV/c/FTC/TAF	No
27	Male	34	Spain	30.1	DTG/RPV	17.0	7260	Wild type/B	E138A/K; Q148R	K101E	DRV/c/FTC/TAF	Yes
28	Male	41	Spain	25.7	DTG/3TC	13.8	563	Not available	No	No	BIC/FTC/TAF	No

VF was defined as 2 consecutive HIV-1 RNA values ≥200 copies/mL or 1 value ≥500 copies/mL prompting treatment discontinuation. Both VF cases with baseline RAMs had M184V; 1 also had K103N.

Abbreviations: 3TC, lamivudine; ABC, abacavir; ART, antiretroviral therapy; BIC, bictegravir; DOR, doravirine; DRV/c, darunavir/cobicistat; DRV/r, darunavir/ritonavir; DTG, dolutegravir; FTC, emtricitabine; INSTI, integrase strand transfer inhibitor; NA, not available; NNRTI, nonnucleoside reverse transcriptase inhibitor; RAL, raltegravir; RAMs, resistance-associated mutations; RPV, rilpivirine; TAF, tenofovir alafenamide; TDF, tenofovir disoproxil fumarate; VF, virological failure; VL, viral load.

^a^Participants without clinically relevant INSTI resistance-associated mutations, for whom a second-generation INSTI-based regimen could have been considered according to the complete resistance profile and clinical context.

All VF cases received an INSTI-containing regimen immediately before CAB + RPV LA. The most common were bictegravir/emtricitabine/tenofovir alafenamide (11/28, 39.3%), dolutegravir/rilpivirine (8/28, 28.6%), and dolutegravir/lamivudine (7/28, 25.0%).

The median time to VF-related discontinuation was 9.1 months (IQR, 7.1–13.3; range, 0.9–31.5). Twenty events (71.4%) occurred by month 12—3 within 6 months and 17 from >6 through 12 months, and 8 (28.6%) occurred later (Figure 2). Supplementary Figure 1 shows reason-specific curves in the strict label-concordant subgroup.

**Figure 2.**
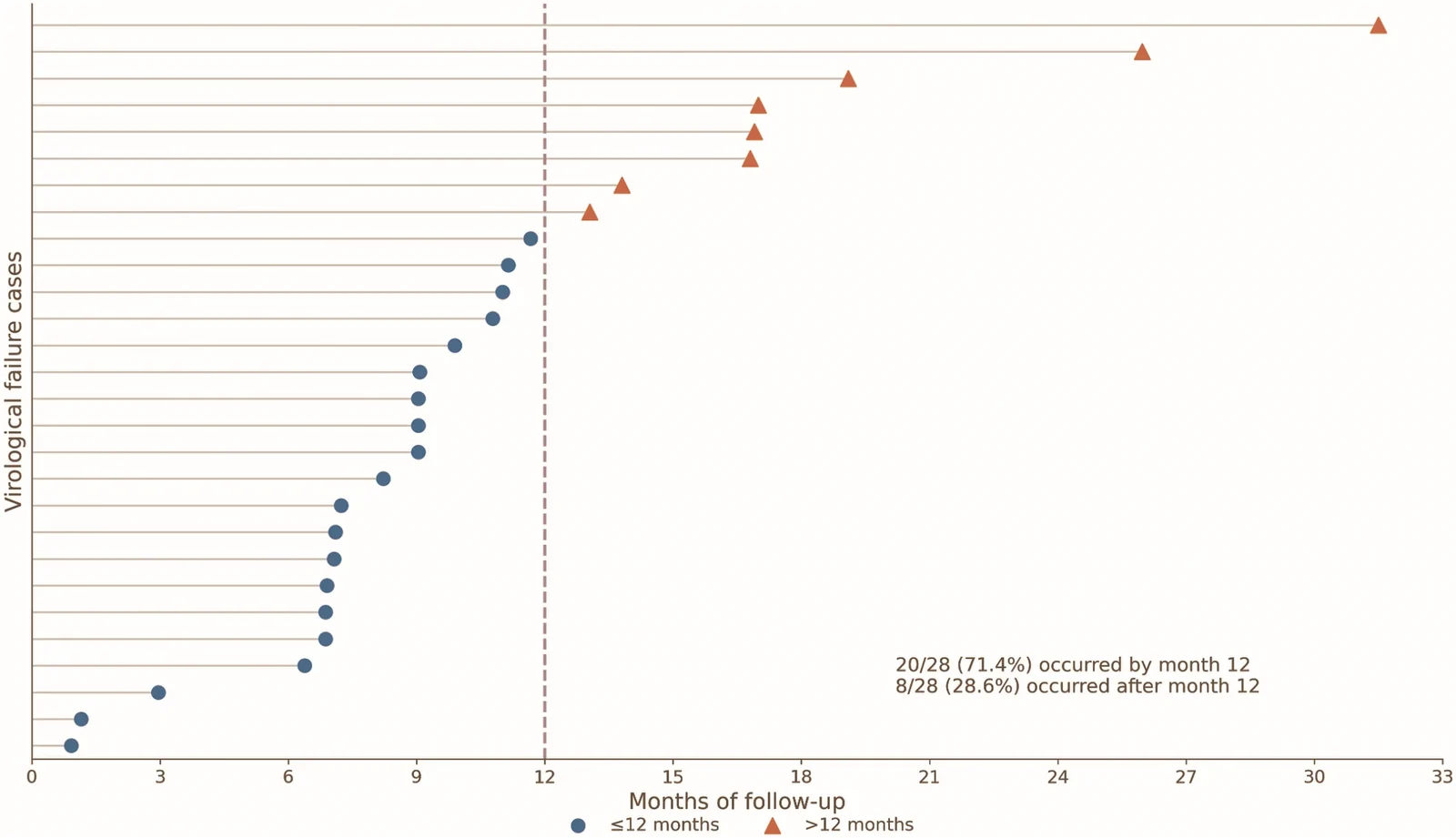
Timing of virological failure (VF) events during follow-up with long-acting cabotegravir plus rilpivirine. Circles indicate VF events occurring by month 12, triangles indicate VF events occurring after month 12, and the dashed vertical line indicates month 12. CAB + RPV LA, long-acting injectable cabotegravir plus rilpivirine.

### Prior Virological History

Previous VF on any ART was documented in 108/3349 (3.2%), 2598 (77.6%) had none, and 643 (19.2%) had missing history. With only 28 events, analyses were not designed to identify independent VF predictors.

### Genotypic Resistance Profiles

Historical genotype availability was ascertainable for 3235/3349 (96.6%); 114 lacked this field. Results were available for 1540/3235 (47.6%), or 46.0% of the cohort. Among these 1540, nucleoside reverse transcriptase inhibitor and NNRTI RAMs were each present in 123 (8.0%), INSTI RAMs in 12 (0.8%), and no RAMs in 1319 (85.6%).

At baseline, 14/28 VF cases were wild type, 12 had missing or noninformative genotypes, and 2 had RAMs: both M184V, with K103N in one. Neither mutation pattern was expected to compromise CAB + RPV under the eligibility rules. At VF, new clinically relevant INSTI RAMs occurred in 16/28 (57.1%), NNRTI RAMs in 14/28 (50.0%), either class in 19/28 (67.9%; 0.57% of the cohort), and both classes in 11/28 (39.3%; 0.33% of the cohort).

Common emergent pathways involved INSTI positions Q148, N155, G140, L74, and E138, and NNRTI positions K101, K103, E138, Y181, and M230; accessory or low-impact polymorphisms were excluded.

### Injection Adherence in Virological Failure Cases

Among VF cases, 27/28 (96.4%) had PDC 100%, and 1/28 (3.6%) had 90%–99.9%; none had <90%. Six participants (21.4%) had 1 recorded delayed injection interval beyond the predefined dosing window, 22 (78.6%) had none, and no participant had ≥2 (Table 3). Thus, VF occurred despite high overall PDC, although a single delayed interval was documented in approximately one-fifth of cases, and PDC does not measure injection technique or drug exposure.

**Table 3.** Injection-Timing Adherence Among Individuals With Virological Failure During Long-acting Cabotegravir Plus Rilpivirine Treatment

Characteristic	Virological Failure Cases (N = 28)
Proportion of days covered, n (%)	
100%	27/28 (96.4%)
90%–99.9%	1/28 (3.6%)
<90%	0/28 (0.0%)
Missing	0/28 (0.0%)
Recorded injections outside the predefined dosing window, n (%)	
0 delays	22/28 (78.6%)
1 delay	6/28 (21.4%)
≥2 delays	0/28 (0.0%)
Missing	0/28 (0.0%)

Data are n/N (%). PDC was calculated from scheduled and administered injection dates using the predefined ±7-d dosing window. Delayed intervals were counted separately from the PDC category.

Abbreviation: PDC, proportion of days covered.

### Outcomes Following Virological Failure

All 28 VF cases switched to oral ART (Table 2): 17 (60.7%) received protease inhibitor–based regimens, principally darunavir-based; 8 (28.6%) received bictegravir/emtricitabine/tenofovir alafenamide; and 3 received other regimens.

At database lock, 24/28 (85.7%) had resuppressed. The other 4 had failed near the cutoff and had limited postswitch follow-up.

### Immunological Outcomes

At baseline, median CD4+ T-cell count was 797 cells/mm^3^ (IQR, 601.5–1030.5; n = 3059), CD4 percentage was 35.0% (IQR, 29.0–41.2; n = 2829), and CD4/CD8 ratio was 1.0 (IQR, 0.7–1.3; n = 3006). Paired analyses showed stable CD4+ counts, modest CD8+ declines, and small CD4/CD8 ratio increases at 12 and 24 months and at the last assessment (Supplementary Table 2). Although some comparisons were statistically significant, the changes were minimal.

Because 36-month paired data were sparse (n = 23 for CD4+ T-cell count; n = 22 for CD8+ T-cell count and CD4/CD8 ratio), this landmark was omitted from Supplementary Table 2.

### Safety and Tolerability

Treatment-limiting tolerability events were infrequent: injection-site reactions affected 79/3349 (2.4%) and systemic adverse events 54/3349 (1.6%). These data cover only events prompting bridging, interruption, or discontinuation and cannot estimate the overall adverse event incidence or exclude new safety signals. Incident pregnancies were not captured separately from pregnancy intention when either prompted treatment change and therefore could not be reliably enumerated.

## DISCUSSION

After a median of 25.1 months, CAB + RPV LA maintained high virological suppression and persistence in this large routine-care cohort. These findings extend trial evidence from ATLAS, FLAIR, SOLAR, CARES, LATTE-2, and ATLAS-2M and are consistent with early implementation cohorts [1–8, 21].

Most participants remained on treatment, and permanent discontinuation was predominantly nonvirological. Persistence in practice depends not only on efficacy and tolerability but also on scheduled-care capacity, patient preference, transfer, and logistics. Some people classified as transferred, relocated, or lost to follow-up may have continued injections elsewhere; therefore, these categories should not be interpreted uniformly as treatment cessation.

Virological failure was uncommon and clustered early: 20/28 events occurred by month 12, most between months 6 and 12. This supports close early viral load follow-up but does not establish a biological mechanism or exclude late VF. Potential contributors include unrecognized resistance, subtype, body composition, injection technique, and pharmacokinetic variability [4, 10, 11, 15, 22]. Therapeutic drug monitoring may help in selected higher-risk or research settings, but routine use remains unsettled because validated targets and prospective evidence of outcome benefits are limited [23–25].

Overall PDC was high among VF cases, although 6 participants had 1 delayed injection interval; therefore, delayed administration was not documented in most failures. Proportion of days covered does not capture needle selection, gluteal delivery, absorption, drug interactions, or interindividual exposure. Long-acting treatment changes the adherence problem rather than eliminating it [26–31].

Emergent INSTI and/or NNRTI resistance occurred in 67.9% of VF cases, but only 0.57% of the cohort; dual-class resistance occurred in 39.3% of VF cases and 0.33% overall. These complementary denominators are essential: resistance was frequent within rare failures but uncommon across treated participants. The pathways were consistent with established patterns [16, 17]. The partially concordant OPERA and CONSENSUS-LAI definitions facilitate comparison while preserving the study's prespecified threshold [12, 18].

Most VF cases resuppressed after resistance-guided oral ART, often with darunavir-based regimens. Therefore, rescue treatment should be individualized to the complete genotype; Q148-based pathways may restrict INSTI activity, whereas preserved predicted susceptibility may permit second-generation INSTI regimens [32].

Low rates of treatment-limiting adverse events support acceptable treatment-limiting tolerability, but non–treatment-limiting events were not systematically collected, and the overall safety incidence cannot be inferred [2, 4, 9].

Strengths include a large nationwide sample, extended follow-up, centralized data capture, and case-level VF and resistance characterization. Limitations include an uncontrolled observational design, clinician-selected treatment, protocol exclusion of pregnancy or breastfeeding and HBsAg positivity, incomplete historical genotypes, noncentral laboratory monitoring, no pharmacokinetic data, PDC's inability to measure injection delivery or exposure, incomplete ascertainment after transfer, relocation, or loss to follow-up, and nonsystematic capture of non–treatment-limiting adverse events and incident pregnancy. Only 28 VF events precluded robust risk-factor inference. Temporarily excluding 5 incompletely updated centers may have reduced partial-follow-up bias but could limit generalizability.

## CONCLUSIONS

In routine care, CAB + RPV LA was associated with high suppression and persistence and few treatment-limiting adverse events over more than 2 years. Virological failure was rare and clustered mainly within 12 months but frequently involved emergent INSTI and/or NNRTI resistance when it occurred.

These findings support CAB + RPV LA as a durable maintenance option for appropriately selected PWH and emphasize early monitoring, prompt confirmation of rebound, resistance testing, and individualized rescue therapy. Further studies should clarify the determinants of early VF.

## Supplementary Material

ofag622_Supplementary_Data

## Supplementary Data

Supplementary materials are available at *Open Forum Infectious Diseases* online. Consisting of data provided by the authors to benefit the reader, the posted materials are not copyedited and are the sole responsibility of the authors, so questions or comments should be addressed to the corresponding author.
