# Supplementary material for: Long-term Effectiveness, Treatment Persistence, and Timing of Virological Failure With Long-Acting Cabotegravir Plus Rilpivirine in Real-World HIV Care: RELATIVITY Cohort
Source: Open Forum Infect Dis. 2026 Oct 21;13(10):ofag622. doi: 10.1093/ofid/ofag622 (PMC13683463; doi:10.1093/ofid/ofag622)
Supplement: Supplementary file 1 [file ofag622_supplementary_data.docx]

**Supplementary Table 1. Baseline patient demographic and clinical characteristics in the strict on-label sensitivity subgroup.**

| **Variables** | **Strict on-label cohort (N = 3,177)** |
| --- | --- |
| Age, years | 45.0 (37.0–54.0) [n=3,177] |
| Body mass index, kg/m² | 25.4 (23.3–28.2) [n=2,725] |
| **Gender, n (%)** |  |
| Female | 474/3,177 (14.9%) |
| Male | 2,680/3,177 (84.4%) |
| Transgender male | 0/3,177 (0.0%) |
| Transgender female | 23/3,177 (0.7%) |
| **Nationality, n (%)** |  |
| Spanish | 2,208/3,167 (69.7%) |
| Other | 959/3,167 (30.3%) |
| **Country/region of birth among foreign-born individuals, n (%)** |  |
| Africa | 66/981 (6.7%) |
| Asia | 9/981 (0.9%) |
| Central Europe | 23/981 (2.3%) |
| Eastern Europe | 43/981 (4.4%) |
| Other Europe | 13/981 (1.3%) |
| South/Central/North America | 773/981 (78.8%) |
| Western Europe | 54/981 (5.5%) |
| **HIV transmission route, n (%)** |  |
| MSM | 1,797/3,177 (56.6%) |
| Heterosexual | 538/3,177 (16.9%) |
| IDU | 201/3,177 (6.3%) |
| Vertical | 22/3,177 (0.7%) |
| Other | 201/3,177 (6.3%) |
| Not available | 418/3,177 (13.2%) |
| CD4 nadir, cells/mm³ | 340.0 (199.0–500.0) [n=2,530] |
| Baseline CD4+ T-cell count, cells/mm³ | 799.0 (606.0–1,031) [n=2,898] |
| Baseline CD4+ T-cell percentage, % | 35.0 (29.0–41.3) [n=2,670] |
| Baseline CD8+ T-cell count, cells/mm³ | 826.0 (606.0–1,106.4) [n=2,845] |
| Baseline CD4/CD8 ratio | 1.0 (0.7–1.3) [n=2,845] |
| HIV-1 RNA at diagnosis, copies/mL | 53,300.0 (14,200.0–181,970.0) [n=2,145] |
| Time from diagnosis to start of first ART, months | 2.0 (0.5–19.6) [n=2,943] |
| **History of AIDS, n (%)** |  |
| Yes | 379/3,177 (11.9%) |
| No | 2,475/3,177 (77.9%) |
| Not available | 323/3,177 (10.2%) |
| Time from start of ART to switch, years | 9.0 (5.0–15.0) [n=3,078] |
| Duration of virological suppression before switch, months | 82.0 (35.0–132.0) [n=2,506] |
| **Previous virological failure on any ART regimen, n (%)** |  |
| Yes | 51/3,177 (1.6%) |
| No | 2,499/3,177 (78.7%) |
| Not available | 627/3,177 (19.7%) |
| **Third drug involved in the virological failure, n (%)** |  |
| INSTI | 0/51 (0.0%) |
| NNRTI | 0/51 (0.0%) |
| PIs | 31/51 (60.8%) |
| Not available | 20/51 (39.2%) |
| **Number of blipsᵃ in the previous 5 years, n (%)** |  |
| 0 | 2,394/2,907 (82.4%) |
| 1 | 318/2,907 (10.9%) |
| 2 | 97/2,907 (3.3%) |
| 3 | 40/2,907 (1.4%) |
| >3 | 58/2,907 (2.0%) |
| Baseline genotype before switch, n (%) | 1,385/3,063 (45.2%) |
| **HIV-1 subtype among those with baseline genotype, n (%)** |  |
| Subtype B | 624/1,385 (45.1%) |
| Subtype A1/A2 | 23/1,385 (1.7%) |
| Subtype F/CRF | 39/1,385 (2.8%) |
| Other subtypes | 59/1,385 (4.3%) |
| Not available | 640/1,385 (46.2%) |
| Wild-type virus without mutations, n (%) | 1301/1,385 (93.9%) |
| Virus harboring RAMs to NRTI, n (%) |  |
| Any NRTI RAM | 84/1,385 (6.1%) |
| M184V | 11/1,385 (0.8%) |
| Others | 76/1,385 (5.5%) |
| Virus harboring RAMs to NNRTI, n (%) |  |
| Any NNRTI RAM | 0/1,385 (0.0%) |
| K103N | 0/1,385 (0.0%) |
| E138A | 0/1,385 (0.0%) |
| Others | 0/1,385 (0.0%) |
| Virus harboring RAMs to INSTI, n (%) |  |
| Any INSTI RAM | 0/1,385 (0.0%) |
| L74M/I/F | 0/1,385 (0.0%) |
| T97A | 0/1,385 (0.0%) |
| Others | 0/1,385 (0.0%) |
| **Number of resistance mutations, n (%)** |  |
| No mutations detected | 1,301/1,385 (93.9%) |
| 1 mutation detected | 84/1,385 (6.1%) |
| 2 mutations detected | 0/1,385 (0.0%) |
| 3 mutations detected | 0/1,385 (0.0%) |
| **Previous oral antiretroviral regimen before switch, n (%)** |  |
| INSTI-based | 2,457/3,177 (77.3%) |
| NNRTI-based | 346/3,177 (10.9%) |
| PI-based | 151/3,177 (4.8%) |
| Other regimens | 103/3,177 (3.2%) |
| Not available | 120/3,177 (3.8%) |
| **Reasons for switching to LA CAB + RPV, n (%)** |  |
| Toxicity | 37/3,177 (1.2%) |
| Drug interaction | 9/3,177 (0.3%) |
| Simplification | 751/3,177 (23.6%) |
| Comfort/quality of life | 1,603/3,177 (50.5%) |
| Malabsorption | 37/3,177 (1.2%) |
| Swallowing disorders | 12/3,177 (0.4%) |
| Patient request | 1,183/3,177 (37.2%) |
| Other reasons for change | 299/3,177 (9.4%) |
| Oral lead-in before injectable treatment, n (%) | 392/3,177 (12.3%) |
| Follow-up time, months | 25.1 (19.3–30.6) [n=3,172] |
| **Effectiveness** |  |
| Last VL≥50 copies/mL (closest VL evaluation to March 1st 2026) | 139/3,177 (4.4%) |
| **Treatment status at end of follow-up, n (%)** |  |
| Temporary oral bridging or interruption | 64/3,177 (2.0%) |
| Permanent discontinuation | 351/3,177 (11.0%) |
| Persistence on treatment | 2,826/3,177 (89.0%) |
| **Reasons for treatment discontinuation or interruption, n (%)** |  |
| Systemic adverse events | 50/3,177 (1.6%) |
| Injection-site reactions | 75/3,177 (2.4%) |
| Virological failure | 26/3,177 (0.8%) |
| Other reasons | 238/3,177 (7.5%) |
| Injection adherence (PDC) ≥90%, among evaluable participants, n/N (%) | 3,090/3,112 (99.3%) |
| Injection-schedule data not evaluable, n/N (%) | 65/3,177 (2.0%) |

Data are median (IQR) unless otherwise stated. ART, antiretroviral therapy; CAB, cabotegravir; IDU, injection drug use; INSTI, integrase strand transfer inhibitor; LA, long-acting; MSM, men who have sex with men; NNRTI, non-nucleoside reverse transcriptase inhibitor; NRTI, nucleoside reverse transcriptase inhibitor; PDC, proportion of days covered; PI, protease inhibitor; RAMs, resistance-associated mutations; RPV, rilpivirine. Blips were HIV-1 RNA values of 50–<200 copies/mL followed by resuppression. The strict on-label subgroup excluded historical NNRTI/INSTI RAMs and prior VF involving those classes. Reasons for switching and discontinuation/interruption could overlap. Mutation-specific rows may overlap. Injection adherence required complete scheduled–administered injection-date pairs.

**Supplementary Table 2. Paired changes in immunological parameters from baseline during follow-up.**

| **Parameter** | **Timepoint** | **Paired N** | **Baseline, median (IQR)** | **Follow-up, median (IQR)** | **Absolute change, median (IQR)** | P value |
| --- | --- | --- | --- | --- | --- | --- |
| CD4+ T-cell count, cells/mm³ | 12 months | 797 | 781 (594–1,000) | 764 (605–989) | -1 (-126–115) | 0.476 |
| CD4+ T-cell count, cells/mm³ | 24 months | 881 | 771 (588–1,010) | 765 (589–969) | -10 (-138.2–102) | 0.054 |
| CD4+ T-cell count, cells/mm³ | 36 months | 23 | 797 (660.5–1,009) | 755 (646–887) | -8 (-77.5–99) | 0.939 |
| CD4+ T-cell count, cells/mm³ | Last available follow-up | 2,992 | 797 (600.9–1,028) | 770 (594–999) | -7 (-138–117) | 0.004 |
| CD8+ T-cell count, cells/mm³ | 12 months | 785 | 837 (602–1,120) | 785 (570–1,045) | -43 (-180–87) | <0.001 |
| CD8+ T-cell count, cells/mm³ | 24 months | 870 | 808 (592.2–1,112) | 731 (542.8–970) | -70.5 (-214–59) | <0.001 |
| CD8+ T-cell count, cells/mm³ | 36 months | 22 | 845 (736–1,007) | 752 (609–970) | -80 (-212.2–14.8) | 0.060 |
| CD8+ T-cell count, cells/mm³ | Last available follow-up | 2,941 | 825.6 (606–1,104) | 753 (551–1,012) | -60 (-214–75) | <0.001 |
| CD4/CD8 ratio | 12 months | 783 | 0.9 (0.7–1.3) | 1.0 (0.7–1.3) | 0.0 (-0.1–0.1) | <0.001 |
| CD4/CD8 ratio | 24 months | 869 | 1.0 (0.7–1.3) | 1.0 (0.8–1.4) | 0.1 (0.0–0.2) | <0.001 |
| CD4/CD8 ratio | 36 months | 22 | 1.1 (0.8–1.4) | 1.1 (0.9–1.4) | 0.1 (0.0–0.2) | 0.080 |
| CD4/CD8 ratio | Last available follow-up | 2,941 | 1.0 (0.7–1.3) | 1.0 (0.8–1.4) | 0.1 (-0.1–0.2) | <0.001 |

Data are median (IQR). Absolute change was follow-up minus baseline. Only participants with baseline and follow-up data were included. P values are from Wilcoxon signed-rank tests. The 36-month landmark was omitted because paired data were sparse (n=23 for CD4+ T-cell count and n=22 for CD8+ T-cell count and CD4/CD8 ratio).

**Supplementary Figure 1. Kaplan–Meier estimates of event-free survival for discontinuation-related outcomes in the strict on-label sensitivity subgroup.**

Panels show systemic adverse events, injection-site reactions, virological failure, and other reasons. Risk sets are event-specific, categories may overlap, and curves are descriptive rather than competing-risk estimates. Of 238 participants with an “other reason,” 233 had evaluable event times within 36 months.

Alt text. Four Kaplan–Meier event-free survival panels over 36 months for systemic adverse events, injection-site reactions, virological failure, and other discontinuation-related reasons in the strict on-label subgroup (N=3,177), with event-specific numbers at risk and interval event counts beneath each panel.
